# Donor–acceptor random regioregular π-conjugated copolymers based on poly(3-hexylthiophene) with unsymmetrical monothienoisoindigo units†

**DOI:** 10.1039/d0ra03557b

**Published:** 2020-05-19

**Authors:** Kaoru Uegaki, Kazuhiro Nakabayashi, Shin-ichi Yamamoto, Toshio Koizumi, Shotaro Hayashi

**Affiliations:** Department of Applied Chemistry, National Defence Academy 1-10-20 Hashirimizu Yokosuka Kanagawa 239-8686 Japan; Graduate School of Organic Materials Science, Yamagata University 4-3-16 Jonan Yonezawa Yamagata 992-8510 Japan; Research Center for Molecular Design, School of Environmental Science and Engineering, Kochi University of Technology 185 Miyanokuchi Kami Kochi 782-8502 Japan hayashi.shotaro@kochi-tech.ac.jp

## Abstract

Donor–acceptor π-conjugated random copolymers based on regioregular poly(3-hexylthiophene), rr-P3HT, with unsymmetrical monothienoisoindigo moieties were obtained by direct arylation polycondensation of 2-bromo-3-hexylthiophene with unsymmetrical monothienoisoindigo motifs under the optimized conditions [palladium-immobilized on thiol-modified silica gel with chloride counter anions, PITS-Cl (2.5 mol%), PivOH (1.0 equiv.), K_2_CO_3_ (3.0 equiv.), DMAc, 100 °C, 24 h]. Incorporation of unsymmetrical monothienoisoindigo electron-acceptor units into the polymers tuned their highest occupied and lowest unoccupied molecular orbital levels, which were close to those of the hole transport material (PEDOT) and electron transport material (PCBM), respectively, in thin-film organic solar cells. Alkyl chains of the unsymmetrical monothienoisoindigo units in the polymers tuned their macrostructural order, resulting in the observation of crystalline patterns and specific absorption peaks in thin films. An organic solar cell containing the most crystalline random copolymer showed an efficiency of 1.91%.

## Abstract 2

Donor–acceptor π-conjugated random copolymers based on regioregular poly(3-hexylthiophene) with unsymmetrical monothienoisoindigo moieties were obtained by direct arylation polycondensation.

## Graphical abstract

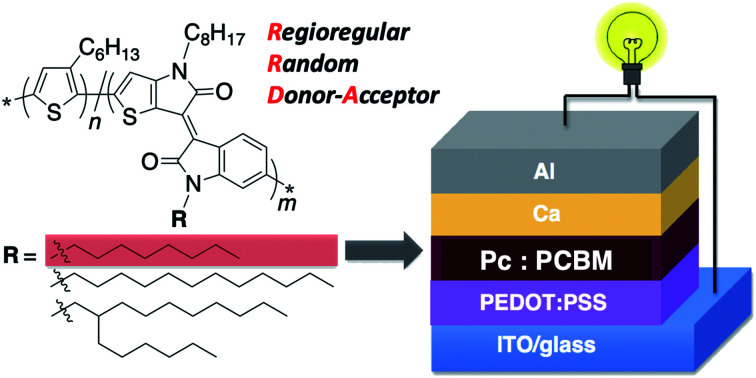

## Introduction

There have been many reports on electron-donating π-conjugated polymers with thiophene units in the main chain.^1^ In particular, regioregular or regiorandom poly(3-hexylthiophene) (rr-P3HT or ra-P3HT) has been synthesized by oxidation^2^ and metal-catalyzed cross-coupling reaction methods (Suzuki–Miyaura cross coupling, Stille cross coupling, and Grignard metathesis).^3^ Meanwhile, although numerous electron-accepting π-conjugated polymers have been synthesized using various electron-accepting aromatic building blocks as monomers, there are not many examples of regioregular electron-accepting π-conjugated polymers.^4^ Recently, regioregular electron-accepting π-conjugated polymers have been synthesized by direct arylation polycondensation using unsymmetrical monothienoisoindigo as a monomer.^5^ The direct arylation reaction can be cross-coupled by directly activating C–H bond of thiophene moiety.^6^ Therefore, the polycondensation of π-conjugated polymers has many advantages. In particular, monomers can be obtained without going through a reaction process to generate anions, such as borylation and stannylation for aromatic skeletons.^7^ The use of direct arylation polycondensation increases the diversity of π-conjugated polymers.^8^

In many cases, rr-P3HT can be obtained by Grignard metathesis polymerization.^3*a*,*b*^ In addition, Suzuki–Miyaura and Stille cross-coupling reactions are also useful approaches to synthesize rr-P3HT.^3*c*,*d*^ Recently, Ozawa's group achieved direct arylation polycondensation of 2-bromo-3-hexylthiophene to form rr-P3HT with high molecular weight.^9^ Lemaire's group reported the first direct arylation polycondensation of 2-bromo-3-alkylthiophene, but the molecular weight of the obtained polymers was low.^10^ Optimization of the polycondensation conditions is important. We and other groups have also synthesized rr-P3HT by direct arylation polycondensation under various conditions.^11^

**Scheme 1:**
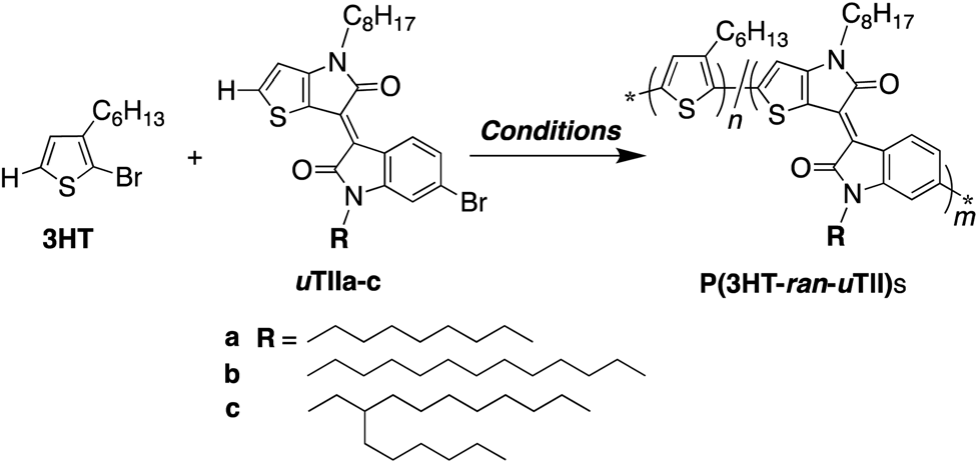
Direct arylation polycondensation of 3HT with uTIIa–c. Conditions: PITS-Cl (1.0–5.0 mol%), PivOH (1.0 equiv.), K_2_CO_3_ (3.0 equiv.), solvent (0.5 or 1.0 M), 100 or 120 °C, 24 h.

High regioregularity increases the crystallinity of thin films, which generally raises the carrier mobility of electronic devices.^12^ Similarly, it is considered that unsymmetrical monothienoisoindigo units can provide π-conjugated polymers with controlled structure. We thought that the crystallinity and gap between the highest occupied molecular orbital (HOMO) and lowest unoccupied molecular orbital (LUMO) energy levels in rr-P3HT thin films could be controlled by including an isoindigo skeleton that functions as an electron acceptor. Thus, we designed donor–acceptor regioregular π-conjugated random copolymers. In this paper, we report the direct arylation polycondensation of 2-bromo-3-hexylthiophene (3HT) with unsymmetrical monothienoisoindigo (uTII) units to synthesize donor–acceptor regioregular π-conjugated random copolymers (Scheme 1). We investigate the properties of these copolymers to assess their suitability for organic solar cell (OSC) applications.

## Results and discussion

### Direct arylation polycondensation

The uTII monomers were synthesized according to previous reports.^13^ First, to investigate the synthesis conditions of the copolymer, Ozawa's method [Herrmann's catalyst (2.5 mol%), tri(*o*-anisyl)phosphine (10 mol%), PivOH (15 mol%), Cs_2_CO_3_ (1.5 equiv.), toluene, 120 °C, 24 h],^9^ Sommer's method [Pd_2_(dba)_3_·CHCl_3_ (1.0 mol%), PivOH (1.0 equiv.), K_2_CO_3_ (3.0 equiv.), chlorobenzene, 100 °C, 2 h],^14^ our chloride-promoted conditions [PdCl_2_ (5.0 mol%), PivOH (1.0 equiv.), K_2_CO_3_ (3.0 equiv.), *N*,*N*-dimethylacetoamide (DMAc), 100 °C, 24 h],^15^ and the method using our solid supported catalyst [PITS-Cl (2.5 mol%), PivOH (1.0 equiv.), K_2_CO_3_ (3.0 equiv.), DMAc, 100 °C, 24 h]^16^ were employed using 3HT and uTIIa as monomers at a ratio of 9 : 1. PITS-Cl (palladium-immobilized on thiol-modified silica gel chloride counter anion) is suitable solid-supported catalyst for the polycondensation.^16^ The yield was determined from the components insoluble in poor solvents (hexane and methanol) and soluble in chloroform. Ozawa's method yielded a polymer with *M*_n_ = 8700 (*M*_w_/*M*_n_ = 1.7) (29% yield). In contrast, no polymer was obtained using Sommer's method. This is considered to be because their conditions are limited to a system using perylenediimide as a monomer.^14^ Under our chloride-promoted conditions, the progress of the reaction was observed, but insoluble polymers were obtained and their analysis was difficult. This was caused by a side reaction at the β-position of the 3HT unit. Using our solid supported catalyst, a polymer with *M*_n_ = 11 200 (*M*_w_/*M*_n_ = 2.9) was obtained (17% yield). Therefore, here, further optimization was performed using our supported catalyst.

To optimize the conditions, the ratio of 3HT to uTIIa was changed (Table 1, Entries 1–3). However, *M*_n_ of the obtained polymer decreased when the ratio of uTIIa was increased. This indicates that uTII is less reactive than 3HT. Therefore, the polymerization was carried out with a 3HT/uTIIa ratio of 9 : 1 and varying amounts of catalyst (Table 1, Entries 1, 4, and 5). *M*_n_ of the obtained polymer was about 10 000, but no dramatic improvement in yield was observed. When the DMAc concentration was 1.0 M, almost no polymer was obtained (Table 1, Entry 6). *N*-Methylpyrrolidone (NMP) was used as the solvent instead of DMAc because it was thought that the results were affected by the solubility of the polymer, but no improvement was observed (Table 1, Entry 7). The reaction was carried out at 120 °C instead of 100 °C, but both yield and *M*_n_ decreased (Table 1, Entry 8).

**Table 1:** Direct arylation polycondensation of 3HT and uTIIa^a^

Entry	Feed ratio, 3HT/uTIIa	Cat., mol%	Sol., conc. (M)	Temp.,°C	*M*_n_ b ^,^ c	*M*_w_/*M*_n_^b^	Yield^b^, %
1	9 : 1	1.0	DMAc (0.5)	100	10.5	2.8	23
2	8 : 2	1.0	DMAc (0.5)	100	4.3	1.9	25
3	7 : 3	1.0	DMAc (0.5)	100	5.8	2.4	20
4	9 : 1	2.5	DMAc (0.5)	100	11.2	2.9	17
5	9 : 1	5.0	DMAc (0.5)	100	9.5	2.7	17
6	9 : 1	2.5	DMAc (1.0)	100	8.4	3.4	2
7	9 : 1	2.5	NMP (0.5)	100	8.5	2.6	22
8	9 : 1	2.5	DMAc (0.5)	120	6.9	1.9	13

a PITS-Cl, PivOH (1.0 equiv.), K_2_CO_3_ (3.0 equiv.), solvent, 24 h. Note that insoluble organic materials for good solvents were not recovered.

b Insoluble in methanol and hexane. Soluble in chloroform.

c kDa.

**Fig. 1:**
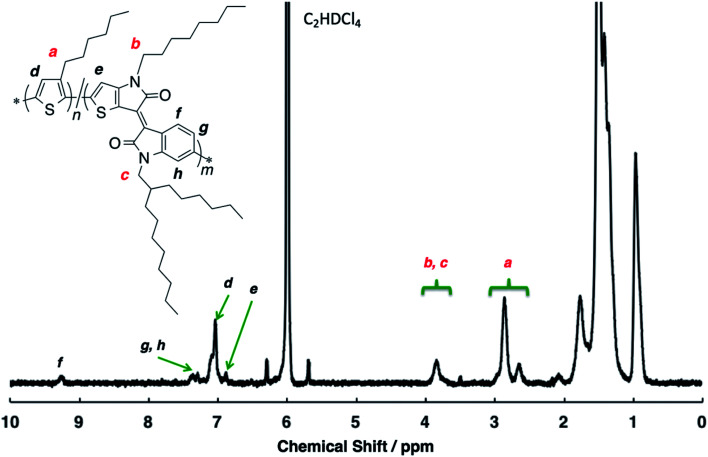
^1^H NMR spectrum of P(3HT-*ran*-uTIIa) in C_2_D_2_Cl_4_ at 100 °C.

The structural analysis of the random copolymer P(3HT-*ran*-uTIIa) was performed by ^1^H NMR spectroscopy measurements (Fig. 1). Side-chain protons were identified as a–c. The ratio of 3HT to uTIIa units was determined from the integral ratio of the methylene proton a of the former and α-methylene protons b and c of the latter. As a result, the ratio of 3HT to uTIIa units was calculated to be 8 : 1 (Table 2). In addition, because the signal of α-methylene protons b and c of uTIIa appeared at 3.7–3.8 ppm, it is considered that only 3HT is adjacent to uTIIa. Focusing on a of the α-methylene proton of 3HT, two signals at 2.6–2.7 and 2.8–2.9 ppm were observed. Therefore, the units adjacent to 3HT were both 3HT and uTIIa. The peak of the aromatic protons in the main chain were identified as d–h (shown in black). Signals were observed from the 3HT units at 6.9–7.1 ppm and uTIIa units at 6.8–6.9, 6.2–6.3, 6.4–6.5, and 9.2–9.3 ppm. Often, a side reaction at the β-position of 3HT to form β-defects is observed. However, the unit ratio calculated from the main chain signal was 8 : 1, which was similar to the result calculated from the side chain. Therefore, it was judged that no β-defects of the thiophene ring were present in the polymer. In P3HT, when the head-to-tail regularity is lost, the integrated value of α-methylene protons of 3HT units appearing at about 2.8–2.9 ppm decreases, and the signal intensity originating from head-to-head regularity at 2.6–2.7 ppm increases. Here, the integration ratio of the signals at 2.8–2.9 and 2.6–2.7 ppm is 8 : 2 and the signal at 2.6–2.7 ppm also includes the 3HT α-methylene proton adjacent to uTIIa. These results indicate that P(3HT-*ran*-uTIIa) is a regular π-conjugated polymer. Thus, it was found that P(3HT-*ran*-uTIIa) is a random copolymer of 3HT and uTIIa.

**Table 2:** Unit ratio and molecular weight of the synthesized polymers^a^

Polymer	Unit ratio^b^	*M*_n_ c ^,^ d	*M*_w_/*M*_n_^c^	Yield^c^, %
P(3HT-*ran*-uTIIa)	8 : 1	11.2	2.9	17
P(3HT-*ran*-uTIIb)	8 : 1	10.8	3.2	13
P(3HT-*ran*-uTIIc)	9 : 1	8.5	2.3	16

a Conditions: PITS-Cl (2.5 mol%), PivOH (1.0 equiv.), K_2_CO_3_ (3.0 equiv.), DMAc, 100 °C, 24 h.

b Estimated by ^1^H NMR spectroscopy.

c Insoluble in methanol and hexane. Soluble in chloroform.

d kDa.

Polymerization was carried out using uTII units with different alkyl chains as a comonomer with our solid supported catalyst, 3HT/uTII (9 : 1), PITS-Cl (2.5 mol%), PivOH (1.0 equiv.), K_2_CO_3_ (3.0 equiv.), and DMAc at 100 °C for 24 h (Table 2). As a result, polymers with *M*_n_ = ∼10 000 were obtained and their unit ratios were approximately the same as the charge ratio.

### Optical properties

**Fig. 2:**
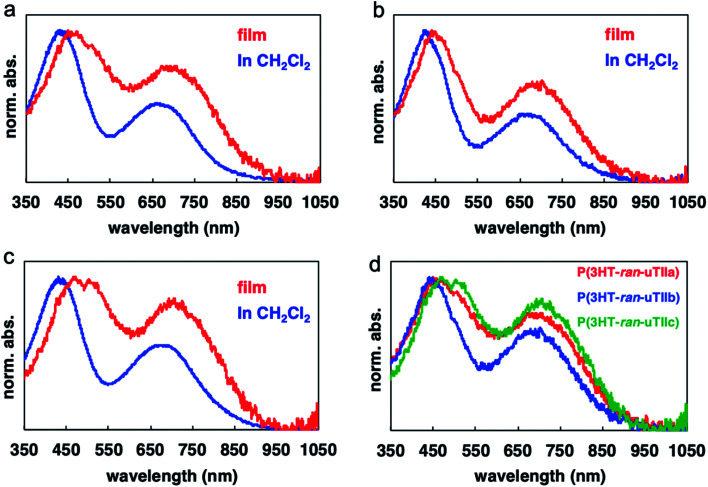
UV-vis absorption spectra of (a) P(3HT-*ran*-uTIIa), (b) P(3HT-*ran*-uTIIb), (c) P(3HT-*ran*-uTIIc) in film and solution states, and (d) all three polymers in the film state.

To investigate the photochemical properties of the polymers, their UV-vis absorption spectra were measured in thin films and dichloromethane solution (Fig. 2 and Table 3). P(3HT-*ran*-uTIIa–c) showed an absorption band derived from charge transfer (CT) interactions, which was not observed in the absorption spectra of rr-P3HT in a thin film and dichloromethane solution. The peak maxima of the absorption bands derived from CT of P(3HT-*ran*-uTIIa–c) appeared at 715, 710, and 720 nm in the thin film and 655, 655, and 660 nm in the dichloromethane solution. That is, an absorption band was observed on the longer wavelength side for the thin film than for the solution. In the rr-P3HT thin film, a specific absorption band associated with the formation of the crystal structure was observed at 600 nm. Similarly, in the thin film of P(3HT-*ran*-uTIIa), which contained crystalline domains, new absorption bands were observed at 510 and 530 nm.

**Table 3:** Absorption peaks (nm) of the polymers

Polymer	In solution		Thin film	
	π–π*	CT	π–π*	CT
P(3HT-*ran*-uTIIa)	440	655	460, 515	715
P(3HT-*ran*-uTIIb)	440	655	450	710
P(3HT-*ran*-uTIIc)	445	660	460, 515	720

### Macrostructural order

**Fig. 3:**
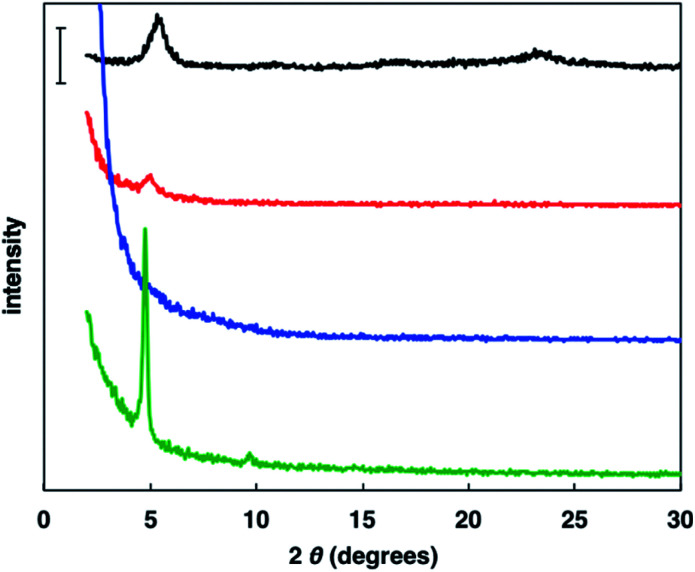
Powder XRD patterns of rr-P3HT (black), P(3HT-*ran*-uTIIa) (red), P(3HT-*ran*-uTIIb) (blue), and P(3HT-*ran*-uTIIc) (green) films. Bar: 1000 counts.

To investigate the crystallinity of the synthesized random copolymer thin films, XRD measurements were conducted.^17^ Generally, rr-P3HT displays a (100) peak originating from its lamellar structure with interdigitation between hexyl side chains and a (010) peak caused by π–π stacking of polythiophene main chain (Fig. 3). The XRD pattern of P(3HT-*ran*-uTIIa) contained a small (100) peak derived from the formation of a lamellar structure with interdigitation. Conversely, no peaks were observed for P(3HT-*ran*-uTIIb). For P(3HT-*ran*-uTIIc), sharp (100) and (200) peaks ascribed to interdigitated packing were observed. The 2*θ* and *d* values of the (100) peak for each polymer were 2*θ* = 5.36° and *d* = 16.5 Å for rr-P3HT, 2*θ* = 4.99° and *d* = 17.7 Å for P(3HT-*ran*-uTIIa), and 2*θ* = 4.76° and *d* = 18.5 Å for P(3HT-*ran*-uTIIc). The *d* value of P(3HT-*ran*-uTIIa, c) was larger than that of rr-P3HT. This result indicates that the introduction of the uTII unit affected the lamellar spacing of the polymer because of the different alkyl chains. P(3HT-*ran*-uTIIc) with an octyl side chain of the uTIIA unit possessed the highest crystallinity of the polymers.

### Electrochemical properties

**Fig. 4:**
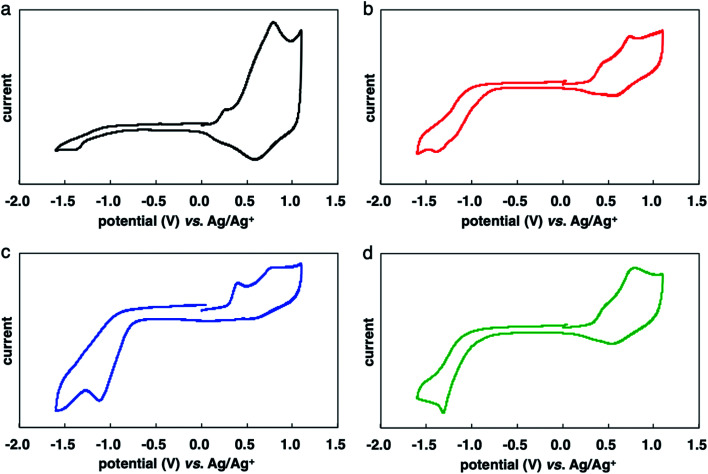
Cyclic voltammograms of rr-P3HT (a: black), P(3HT-*ran*-uTIIa) (b: red), P(3HT-*ran*-uTIIb) (c: blue), and P(3HT-*ran*-uTIIc) (d: green). Conditions: electrolytic solution of 0.1 M Bu_4_NBF_4_/CH_3_CN, working electrode: glassy carbon or ITO/PET, counter electrode: Pt, reference electrode: Ag/AgCl, scan rate: 100 mV s^−1^.

The results of CV measurements of the polymers are shown in Fig. 4. The oxidation potentials of P(3HT-*ran*-uTIIa–c) (about 0.30 V) were slightly lower than that of P3HT (0.32 V). The introduction of the electron-accepting uTII unit lowered the oxidation potential of the polymers. The reduction potentials of P(3HT-*ran*-uTIIa–c) were about −0.80 V because of the introduction of uTII units. Table 4 lists the HOMO and LUMO energy levels of each random copolymer and the corresponding band gaps estimated from the results of electrochemical measurements.^18^ The HOMO values of P(3HT-*ran*-uTIIa), P(3HT-*ran*-uTIIb), and P(3HT-*ran*-uTIIc) were similar at −5.10, −5.09, and −5.12 eV, respectively; their LUMO values were −3.92, −4.03, and −3.80 eV, respectively. The band gaps of the polymers were extremely narrow at 1.18, 1.06, and 1.32 eV for P(3HT-*ran*-uTIIa), P(3HT-*ran*-uTIIb), and P(3HT-*ran*-uTIIc), respectively.

**Table 4:** Electrochemical properties of the polymers

Polymer	HOMO^a^ (eV)	LUMO^a^ (eV)	Electrochemical band gap (eV)
P3HT	−5.12	−3.54	1.58
P(3HT-*ran*-uTIIa)	−5.10	−3.92	1.18
P(3HT-*ran*-uTIIb)	−5.09	−4.03	1.06
P(3HT-*ran*-uTIIc)	−5.12	−3.80	1.32

a Estimated from the onset oxidation and reduction potentials using *I*_p_(HOMO) = −(*E*_onset,ox_ + 4.80) (eV) and *E*_a_(LUMO) = −(*E*_onset,red_ + 4.80) (eV). Energy band gaps were determined from (*E*_a_(LUMO) − *I*_p_(HOMO)) (eV).

The above measurements indicated that the P(3HT-*ran*-uTIIc) film possessed macrostructural order and P(3HT-*ran*-uTIIc) has a narrow band gap and absorbs light over an extremely wide range of the UV-vis region. HOMO and LUMO levels of P(3HT-*ran*-uTIIc) were close to those of PEDOT (HOMO: −4.94 eV) and PCBMs (LUMO: −3.89 eV), respectively, which are carrier transport materials often used in thin-film OSCs (Fig. S6†). We consider that P(3HT-*ran*-uTIIc) is suitable for the active layer of OSCs because of its highly crystalline domains.

### Organic thin-film solar cells

Thin-film OSCs were fabricated using P(3HT-*ran*-uTIIc), which formed the most highly crystalline domains of the polymers (Fig. 3). The OSC structure was ITO/PEDOT:PSS/P(3HT-*ran*-uTIIc):PCBM/Ca/Al. The casting solvent was chlorobenzene and the solution concentration was 30 mg mL^−1^. The weight ratio of P(3HT-*ran*-uTIIc) to PCBM was 2 : 3.

Although the factors that determine the open circuit voltage (*V*_oc_) of OSCs are not fully understood, it has been empirically shown that there is a positive correlation between the HOMO of the donor molecule and LUMO of the acceptor molecule.^19^ The HOMO–LUMO gap was not affected by the operating conditions, so *V*_oc_ was constant at about 0.59 V. In contrast, the short-circuit current density (*J*_sc_) is correlated with the crystallinity of the polymer and the bulk heterostructure of the active layer. Because *J*_sc_ is strongly influenced by the element structure and manufacturing conditions, it is important to examine these factors. In this study, the fabrication conditions were examined in Run 1–3 in Table 5, and the device structure was examined in Run 4. First, in Run 1 and 2, the film formation conditions of 800 rpm/60 s and 450 rpm/60 s were compared. For the OSC containing the film formed at 450 rpm for 60 s, *J*_sc_ was high (5.22 mA cm^−2^). This is probably because the thickness of the film formed by spin coating at 450 rpm for 60 s was appropriate. Next, in Run 2 and 3, we examined the effect of annealing. *J*_sc_ of the OSC containing the annealed film (Run 3) was only 3.28 mA cm^−2^, which might result from morphological change of the active layer in nano scale by annealing. These comparisons revealed that the optimum conditions were those used in Run 2. The power conversion efficiency (PCE) of the OSC produced using a thin film fabricated under these conditions was 1.78%. To further improve PCE, Run 4 used PC_71_BM, which is a stronger acceptor than PC_61_BM. As a result, PCE increased to a maximum of 1.91% (Fig. S7†).

**Table 5:** Performance of OSCs^a^^,^^b^

Run	Conditions (rpm s^−1^)	Annealing (°C min^−1^)	*V*_oc_ (V)	*J*_sc_ (mA cm^−2^)	FF	PCE (%)
1^b^	800/60	—	0.58	4.43	0.60	1.60
2^b^	450/60	—	0.60	5.22	0.57	1.78
3^b^	450/60	150/10	0.59	3.28	0.62	1.20
4^c^	450/60	—	0.58	5.49	0.58	1.91

a ITO/PEDOT:PSS/active layer/Ca/Al architecture.

b The active layer was prepared from a chlorobenzene solution (30 mg mL^−1^) of polymer : PC_61_BM (2 : 3 wt%).

c PC_71_BM was used instead of PC_61_BM (polymer : PC_71_BM ratio = 2 : 3 wt%).

## Conclusions

The random copolymerization of 3HT and uTII by direct arylation polycondensation was conducted to synthesize donor–acceptor regioregular random π-conjugated polymers P(3HT-*ran*-uTIIa–c) with narrow band gaps. We obtained highly crystalline thin films by using an octyl chain in the uTII unit, uTIIc. An organic thin-film solar cell containing P(3HT-*ran*-uTIIc) with highly crystalline domains showed a PCE of 1.91%.

## Experimental

### Chemicals

Commercially available 2-bromo-3-hexylthiophene (3HT, Tokyo Chemical Industry), pivalic acid (PivOH, Tokyo Kasei), potassium carbonate (K_2_CO_3_, Junsei Chemical), and dehydrated *N*,*N*-dimethylacetamide (DMAc, Wako Chemical) were used. The solid phase-supported palladium catalyst (PITS-Cl) was synthesized from 3-mercaptopropyl silica gel (0.5–0.8 mmol g^−1^, Tokyo Kasei) according to our previous report.^16^ Each uTII monomers were synthesized according to the previous report.^13^

### Direct arylation polycondensation

uTIIa (0.050 mmol), PivOH (51 mg, 0.50 mmol), potassium carbonate (212 mg, 1.5 mmol), PITS-Cl (21 mg, 0.013 mmol), and 2-bromo-3-hexylthiophene (111 mg, 0.45 mmol) were added to a vial. The air in the mixture was slowly replaced by flowing argon gas. Dehydrated *N*,*N*-dimethylacetamide (1.0 mL) was added and then the mixture was stirred at 100 °C for 24 h. The reaction mixture was cooled, filtered, and then the filtrate was added dropwise into a large amount of methanol to obtain a bluish black precipitate. The precipitate was washed with acetone and then hexane. The residue was dissolved in chloroform, concentrated, and dried *in vacuo* to obtain a bluish black solid. After washing again with acetone and hexane, Soxhlet extraction was performed using chloroform.

#### P(3HT-*ran*-uTIIa)

Yield: 17%. GPC (PS standard): *M*_n_ = 11 200, *M*_w_/*M*_n_ = 2.9. ^1^H NMR (300 MHz, *δ*, ppm from 1,1,2,2-tetrachloroethane-*d*_2_): 9.3–9.2 (*Ar-H*, br), 7.5–6.8 (*Ar-H*, br), 4.0–3.6 (NC*H*_2_–, br), 3.0–2.5 (*Ar*C*H*_2_–, br), 2.2–1.9 (NCH_2_C*H*(CH_2_–)_2_, br), 1.7–1.1 (methylene, br), 1.1–0.8 (alkyl-C*H*_3_, br).

#### P(3HT-*ran*-uTIIb)

Yield: 13%. GPC (PS standard): *M*_n_ = 10 800, *M*_w_/*M*_n_ = 3.2. ^1^H NMR (300 MHz, *δ*, ppm from 1,1,2,2-tetrachloroethane-*d*_2_): 9.3–9.2 (*Ar-H*, br), 7.5–6.8 (*Ar-H*, br), 4.0–3.6 (NC*H*_2_–, br), 3.0–2.5 (*Ar*C*H*_2_–, br), 2.2–1.9 (NCH_2_C*H*(CH_2_–)_2_, br), 1.7–1.1 (methylene, br), 1.1–0.8 (alkyl-C*H*_3_, br).

#### P(3HT-*ran*-uTIIc)

Yield: 16%. GPC (PS standard): *M*_n_ = 8500, *M*_w_/*M*_n_ = 2.3. ^1^H NMR (300 MHz, *δ*, ppm from 1,1,2,2-tetrachloroethane-*d*_2_): 9.3–9.2 (*Ar-H*, br), 7.5–6.8 (*Ar-H*, br), 4.0–3.6 (NC*H*_2_–, br), 3.0–2.5 (*Ar*C*H*_2_–, br), 2.2–1.9 (NCH_2_C*H*(CH_2_–)_2_, br), 1.7–1.1 (methylene, br), 1.1–0.8 (alkyl-C*H*_3_, br).

### Measurements

Liquid-state ^1^H and ^13^C NMR spectra were recorded on a JEOL EX-300 spectrometer. Molecular weight (*M*_n_) and polydispersity (*M*_w_/*M*_n_) of the polymers were estimated by size exclusion chromatography (SEC, tetrahydrofuran as eluent, polystyrene standards, 40 °C). Ultraviolet-visible (UV-vis) absorption spectra were obtained on an Ocean Optics USB4000-XR1 fiber spectrometer with a DH2000-BAL tungsten halogen light source. Cyclic voltammetry (CV) measurements were performed using an ALS 611 analyzer. A three-electrode system equipped with a glassy carbon, platinum counter electrode, and Ag/AgCl as a reference electrode was used in an electrolyte solution of acetonitrile containing 0.1 M tetraethylammonium tetrafluoroborate. Powder X-ray diffraction (XRD) analysis was performed using a JEOL JDX-3530 X-ray diffractometer. DSC analysis was performed by a Shimadzu DS-60, which measured during heating from room temperature to 400 °C at heating rate of 10 °C min^−1^ in nitrogen. TGA analysis was performed by a Shimadzu TA-60, which measured during heating from room temperature to 600 °C at heating rate of 20 °C min^−1^ in nitrogen.

An example of the fabrication of a thin-film OSC (Run 2 in Table 5) is described below. A poly(3,4-ethylenedioxythiophene):polystyrenesulfonate (PEDOT:PSS) layer with a thickness of 30 nm was spin-coated on an ITO substrate with a surface resistivity of 15 Ω □^−1^ that had previously been cleaned and treated with UV/ozone. The PEDOT:PSS layer was annealed at 140 °C for 20 min. The ITO substrate was moved into a glove box and then a dehydrated chlorobenzene solution of PEDOTDPT:PC_61_BM (PEDOTDPT:PC_61_BM weight ratio = 1 : 2, solution concentration: 12 mg mL^−1^) was spin-coated on the ITO substrate at 4000 rpm for 60 s to form a PEDOTDPT:PC_61_BM blend active layer. A calcium buffer layer (thickness: 30 nm) and aluminum electrode (thickness: 100 nm) were formed by vacuum deposition and then the device was sealed with a UV-curable sealant. The current density (*J*)–voltage (*V*) characteristics of cells were measured using a Keithley 2440 5A source meter under simulated sunlight AM 1.5 G (100 mW cm^−1^) irradiation. The simulated sunlight intensity was corrected using an Oriel P/N 91150V reference cell.

## Conflicts of interest

There are no conflicts to declare.

## Supplementary Material

RA-010-D0RA03557B-s001
