# Supplementary material for: Donor–acceptor random regioregular π-conjugated copolymers based on poly(3-hexylthiophene) with unsymmetrical monothienoisoindigo units
Source: RSC Adv. 2020 May 19;10(32):19034–40. doi: 10.1039/d0ra03557b (PMC9053906; doi:10.1039/d0ra03557b)
Supplement: RA-010-D0RA03557B-s001 [file RA-010-D0RA03557B-s001.pdf]

Electronic Supplementary Material (ESI) for RSC Advances.
This journal is © The Royal Society of Chemistry 2020

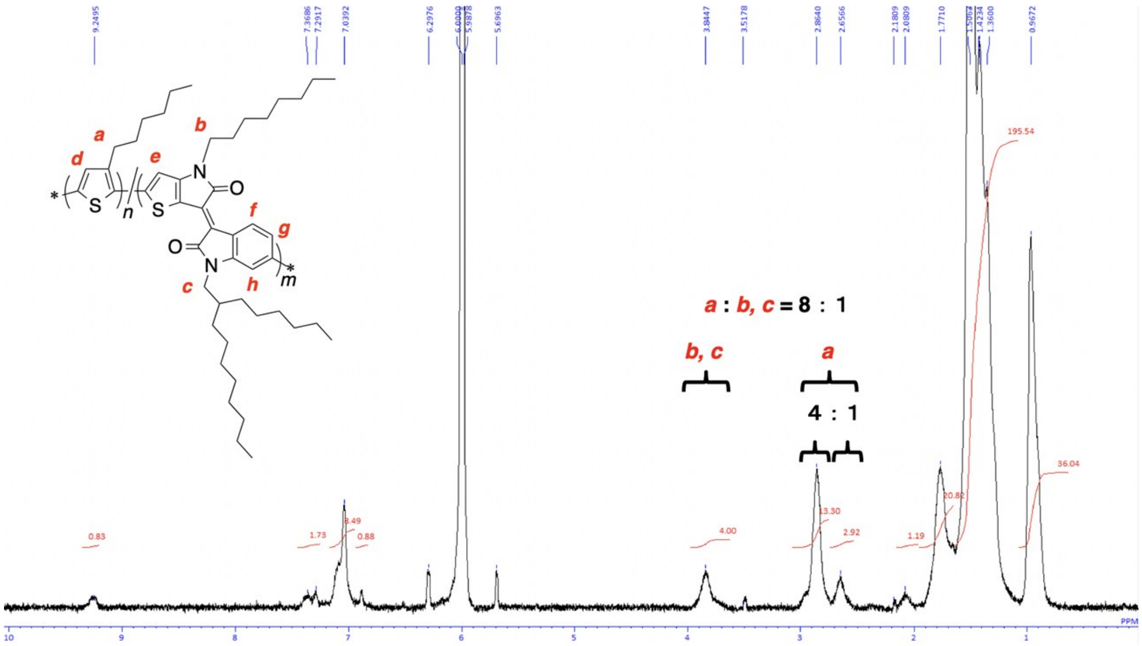

Fig. S1 ^{1}H NMR spectrum of **P(3HT-*ran*-uTIIa)**.

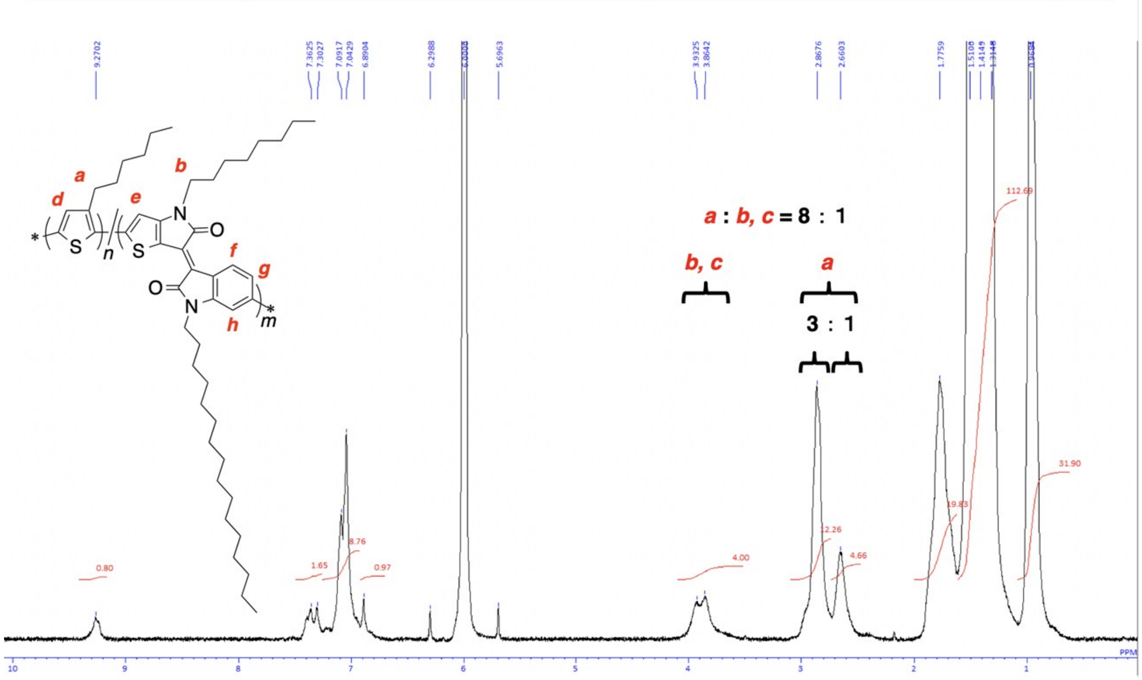

Fig. S2 ^{1}H NMR spectrum of **P(3HT-*ran*-uTIIb)**.

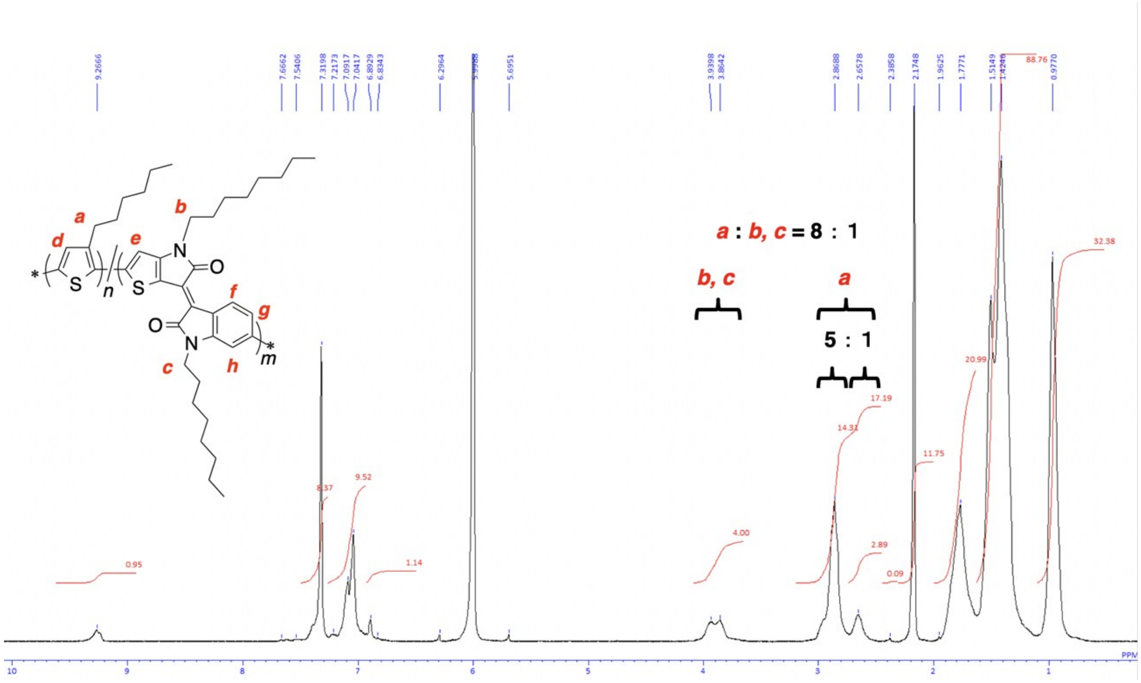

Fig. S3 ^{1}H NMR spectrum of **P(3HT-*ran*-uTIIc)**.

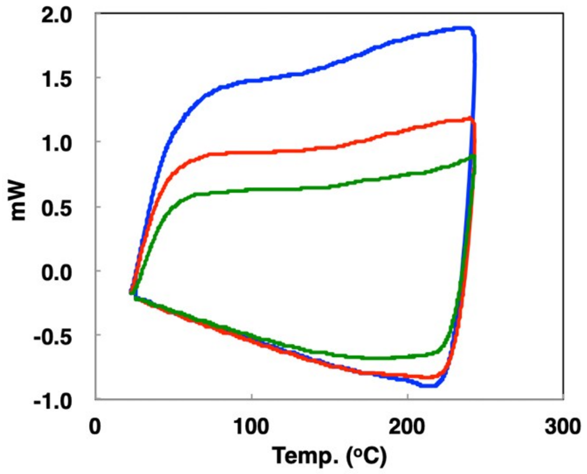

Fig. S4 DSC traces of the polymers.

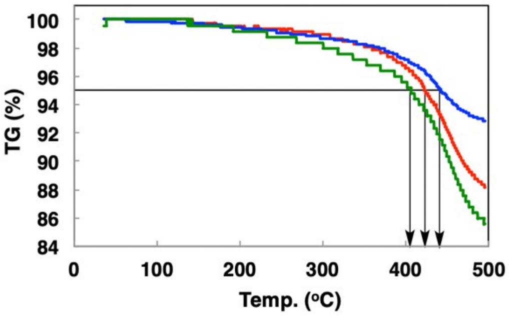

Fig. S5. TGA data of the polymers.

Table S1. Electrochemical properties of the polymers

Polymer	Anodic peaks (V)	Cathodic peaks (V)
**P3HT**	0.31, 0.73	–
**P(3HT-*ran*-uTIIa)**	0.42, 0.69	−1.07
**P(3HT-*ran*-uTIIb)**	0.39, 0.72	−4.03
**P(3HT-*ran*-uTIIc)**	0.48, 0.60	−1.30

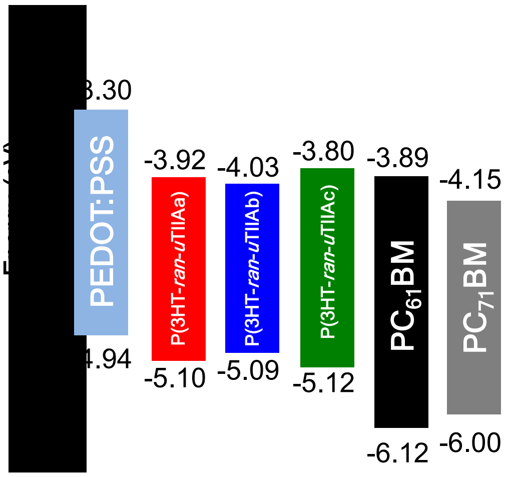

Fig. S6 Energy levels of the polymers.

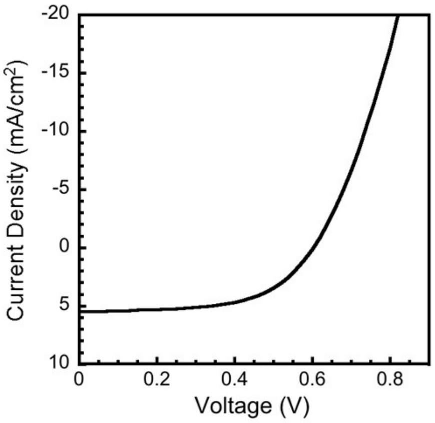

Fig. S7 IV curve of the devices.
